# THE JOINT EFFECTS OF EXPOSURE TO AIR POLLUTION AND HEAT ON EPIGENETIC AGING IN THE HEALTH AND RETIREMENT STUDY

**DOI:** 10.1093/geroni/igae098.0615

**Published:** 2024-12-31

**Authors:** Kristina Dang, Eunyoung Choi, Caleb Finch, Eileen Crimmins, Jennifer Ailshire

**Affiliations:** University of Southern California, Los Angeles, California, United States; University of Southern California, Los Angeles, California, United States; University of Southern California, Los Angeles, California, United States; University of Southern California, Los Angeles, California, United States; University of Southern California, Los Angeles, California, United States

## Abstract

Prior research shows independent associations of exposure to air pollution and heat on epigenetic alterations. However, less is known about the combined effects of long-term air pollution (PM2.5) and acute heat events. We examine joint effects of elevated PM and heat on DNA methylation (DNAm). Data from the 2016 Health and Retirement Study DNAm Sample (N=3,464) were merged at the census tract level to (1) annual ambient concentrations of PM2.5 from CACES and (2) daily heat index data averaged 7-days before blood collection from gridMET. Four categories of joint PM2.5 and heat were analyzed: (1=reference) low PM2.5 (< 9.2 𝜇g/m3) and low heat (< 80 on heat index); (2) low PM2.5 and high heat; (3) high PM2.5 and low heat; and (4) high PM2.5 and high heat. We used five epigenetic aging measures: Horvath, Hannum, PhenoAge, GrimAge, DunedinPACE. Linear models were adjusted for age, sex, cell type, race/ethnicity, education, and neighborhood poverty. Compared to the reference of low PM2.5 and heat, we found associations of short-term heat and low PM with accelerated DNAm aging for Hannum (𝛽=0.61 95%CI:0.05, 1.18) and PhenoAge 𝛽=0.90 95%CI:0.28, 1.52). High PM2.5 and low heat had weaker associations (Hannum 𝛽=0.41 95%CI:-0.28, 1.11; PhenoAge 𝛽=0.13 95%CI:-0.61, 0.89), as did joint effects of high PM2.5 and heat (Hannum 𝛽=0.21 95%CI:-0.61, 1.03; PhenoAge 𝛽=0.27 95%CI:-0.68, 1.22). Short-term heat may accelerate epigenetic aging, particularly in areas with low air pollution. These findings suggest underlying physiological responses to acute heat exposures may differ between residents in low and high air pollution environments.

